# An Optimization Design Method of Rigid-Flexible Soft Fingers Based on Dielectric Elastomer Actuators

**DOI:** 10.3390/mi13112030

**Published:** 2022-11-19

**Authors:** Fuhao Ouyang, Yuanlin Guan, Chunyu Yu, Xixin Yang, Qi Cheng, Jiawei Chen, Juan Zhao, Qinghai Zhang, Yang Guo

**Affiliations:** 1 School of Mechanical and Automotive Engineering, Qingdao University of Technology, Qingdao 266520, China; 2 Key Lab of Industrial Fluid Energy Conservation and Pollution Control, Ministry of Education, Qingdao University of Technology, Qingdao 266520, China; 3 School of Automation, Qingdao University, Qingdao 266017, China; 4 College of Computer Science and Technology, Qingdao University, Qingdao 266017, China

**Keywords:** soft finger, optimization design method, dielectric elastomer, bending angle

## Abstract

The soft gripper has received extensive attention, due to its good adaptability and flexibility. The dielectric elastomer (DE) actuator as a flexible electroactive polymer that provides a new approach for soft grippers. However, they have the disadvantage of having a poor rigidity. Therefore, the optimization design method of a rigid-flexible soft finger is presented to improve the rigidity of the soft finger. We analyzed the interaction of the rigid and soft materials, using the finite element method (FEM), and researched the influence of the parameters (compression of the spring and pre-stretching ratio of the DE) on the bending angle. The optimal parameters were obtained using the FEM. We experimentally verified the accuracy of the proposed method. The maximum bending angle is 19.66°. Compared with the theoretical result, the maximum error is 3.84%. Simultaneously, the soft gripper with three fingers can grasp various objects and the maximum grasping quality is 11.21 g.

## 1. Introduction

Robots have been widely used in agricultural production, industrial collaboration, underwater operations, deep space exploration and other fields. As an important executive component and interactive component of robots, the design of mechanical grippers plays a decisive role on the performance of robots [1,2,3]. The rigid grippers have been widely applied in industry and social life. With the advancement of science and technology in recent years, the disadvantages of rigid grippers have gradually been exposed, such as their heavy weight, complex structure, high rigidity, and vulnerability as fragile objects. Compared with the traditional rigid gripper, the soft gripper [4] has the advantages of a high flexibility, simple mechanical structure, short response time, lightweight and a good bionic performance. Therefore, the soft gripper has gradually gained attention in design and production [5,6].

The soft grippers have been developed, based on different actuators, such as metal composite materials [7,8,9], soft fluid materials [10,11,12], pneumatic soft materials [13,14,15,16] and dielectric elastomer (DE) polymer [17,18]. Li et al. [9] studied a “periodic” soft gripper made of metal composite materials, based on the strain energy storage with a larger bending displacement under the same cross-section condition. However, this kind of metal composite material-driven soft gripper is mainly driven by the shape memory effect of the material, and the driving method is cumbersome. He et al. [10] designed a heating grid structure around the liquid crystal elastomer, which was controlled by an external voltage to realize the driving of the soft gripper. Although the soft fluid-driven grippers have the advantages of the simple driving method, their design structures are relatively complicated. Furthermore, hydrogels with a high stretchability and mechanical compliance also have attracted the intensive interest from many researchers [11]. However, hydrogels have the disadvantage of having a low rigidity. Lotfiani et al. [13] used a soft pneumatic actuator to make a two-finger gripper, which could grasp objects with a weight of about 830 g. Pneumatic soft grippers have the advantages of a high adaptability and strong safety, but they have high requirements for structural sealing, and have disadvantages, such as a low rigidity and complex structure. Witchuda et al. [18] designed a rigid frame and stacked six layers of DE membranes on the frame to prepare a two-finger gripper that could grasp 17 g. In contrast, DE materials not only have the advantages of a large deformation and fast response, but also have similar advantages to natural muscles, in terms of energy density and strain [19,20]. At present, the rigidity is the main problem in the design and manufacture of the soft gripper.

Researchers have carried out a series of studies to solve the rigidity of the grippers by the DE actuators. For example, David et al. [21] used the conductive shape memory polymer as the driving electrode of the gripper and the bending grasp was realized by controlling the temperature of the electrode. However, there are disadvantages, such as a cumbersome driving method and a long response time. Allen et al. [22] proposed the design of a variable rigidity actuator, which can independently adjust the rigidity. However, there are problems, such as a large volume and complex structure. Chen et al. [23] improved the structure of the DE fingers by using load-bearing beams, which increased the rigidity but the bending angle was reduced by 13%. The above studies have improved the rigidity of the DE gripper to a certain extent, but the improved grippers have disadvantages, such as a large volume, slow response speed, and cumbersome driving method. At the same time, there are few design analyses and optimization methods for the soft gripper. Therefore, the design and optimization of the soft gripper structure deserve further research.

Some scholars have studied the theoretical model of the soft gripper, based on the DE membrane. Nguyen et al. [24] characterized the viscoelastic properties of the DE membranes, combined with the Kelvin–Voigt model and the generalized Maxwell model, to describe the deformation process. The deformation process of the model can be predicted through a formula derivation, but the derivation process is complicated and the accuracy is low. It is the absence of structural optimization. However, the finite element method (FEM) avoids the complicated derivation process and obtains more intuitive and accurate results. It has been proven to be an effective modeling and optimization method. Zhou et al. [25] used simulation software to establish a simplified model of the bilayer structure of DEs. The experimental results showed that the simulation results were basically consistent with the experimental data, but there was a large error in the radial stress at both ends. Liu et al. [26] designed a walking machine with a minimum energy structure, based on the DE and the motion was simulated by finite element software. At present, some scholars have designed the configuration of a rolled DEA [1,27,28]. However, there are relatively few optimum structural design methods of soft grippers in related papers, which means that each new structure requires a lot of experimental verifications. In the model construction and analysis process of the above research, the structure is simplified and analyzed. In particular, there are few numerical theoretical studies related to the combined analysis of the DE membranes and other materials. Therefore, the optimal design method of the soft gripper deserves further research.

In order to obtain the high performance of the soft gripper, based on the DE actuator, the optimization design method of the rigid-flexible soft finger are proposed. In Section 2, the soft finger will be designed and the influence of two variables (compression of the spring and pre-stretching ratio of the DE) on the bending performance of the soft fingers will be discussed by the FEM and two optimal parameters will be obtained. In Section 3, the optimization design method will be verified in the experiments, and the optimal soft fingers will be manufactured. In addition, the experimental soft gripper will be tested.

## 2. Structural Design and Theoretical Model Analysis of the Soft Finger

### 2.1. Structural Design of the Soft Finger

**Figure 1:**
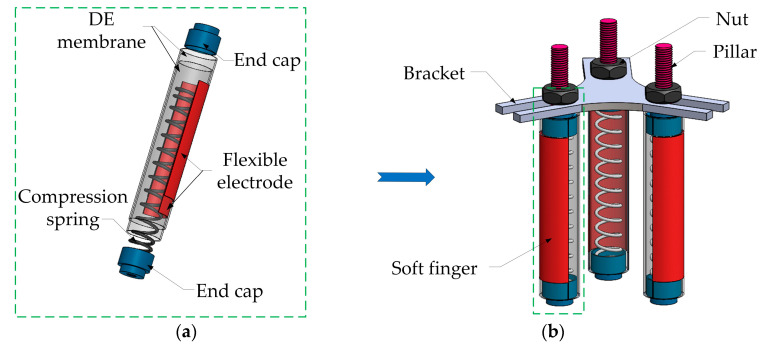
(**a**) Schematic diagram of the soft gripper. (**b**) Schematic diagram of the soft fingers.

In order to enhance the rigidity of the soft finger, based on the DE actuator, the rigid-flexible finger is proposed, as shown in Figure 1a. It is composed of a DE membrane, a spring, a flexible electrode and an end cover. The DE membrane (VHB 4910, initial thickness 1 mm) is used as the actuator, and the spring is applied as the brace structure. In order to achieve the bending performance, the flexible electrode is coated on the inner and outer surfaces of the part of the DE. In order to research the characteristics and application of soft fingers, we propose the soft gripper with three non-collinear fingers, based on the DE actuator, and the schematic diagram is shown in Figure 1b. The fingers are distributed at 120° and fixed by struts and nuts.

### 2.2. Theoretical Basis of the Soft Finger

**Figure 2:**
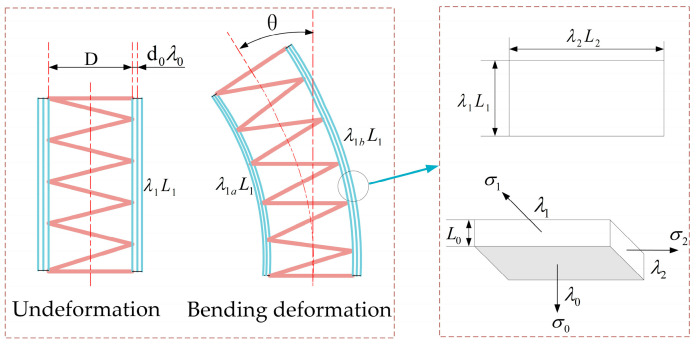
Force analysis diagram of the soft fingers and their membrane.

In order to analyze and optimize the bending properties of the soft finger, we propose the mechanical theoretical model. As shown in Figure 2, according to the hyperelasticity of the DE, the VHBs are equibiaxially pre-stretched for the length and the width, and the excellent range of the pre-stretching ratio is 300%~400%. In order to improve the bending deformation, the actuator is composed of multilayered VHBs, and the spring is compressed to store the energy in the finger. The schematic diagram of the force analysis is shown in Figure 2. The initial size of each DE membrane is $L_{0}\times L_{1}\times L_{2}$. The rates of the DE membrane on the thickness, length and width direction are $\lambda_{0},\;\lambda_{1},\;\lambda_{2}$, respectively.

Due to the DE membranes being incompressible and obtaining an equal biaxial pre-stretching deformation for the length and width, there are $\lambda_{1}=\lambda_{2}=\lambda$ and $\lambda_{0}=\lambda^{-2}$. Once the compressed spring is wined by the pre-stretched DE membrane, combined with the structure, the circumferential length is the same as the circumference of the spring that is

(1) $$\lambda_{1}L_{1}=\lambda_{t}h_{0}$$

(2) $$\lambda_{2}L_{2}=\pi R$$

where $\lambda_{t}$ is the compression rate of the spring, $h_{0}$ is the original length of the spring, and *R* is the outer diameter of the spring.

*X* is set as the electrical displacement of the DE membrane, which can be expressed as

(3) $$X=\frac{Q}{L_{1}L_{2}\lambda }$$

where *Q* is the charge between the electrodes.

The thermodynamics of DEs can be represented by the density of the Helmholtz free energy. In order to apply the hyperelasticity of the DE for the soft finger, we adopt the Ogden model and the free energy density function can be expressed as

(4) $$W\left(\lambda_{1},\lambda_{2},X\right)=\sum_{i=1}^{3}\frac{\mu_{i}}{\alpha_{i}}\left(\lambda_{1}^{\alpha_{i}}+\lambda_{2}^{\alpha_{i}}+\lambda_{1}^{-\alpha_{i}}\lambda_{2}^{-\alpha_{i}}-3\right)+\frac{X^{2}}{2\varepsilon }$$

where *μ*_i_ and *α*_i_ are the material parameters, obtained by the subsequent simulation fitting of the DE membrane; *λ*_1_*, λ*_2_, *λ*_3_ represent the elongation; *ε* is the dielectric constant of the DE membrane.

The free energy of the membrane is equivalent to the electric energy, the stored energy of the compressed spring, and the energy of the inertial force.

(5) $$L_{0}L_{1}L_{2}\delta W=U\delta Q+K\left(h_{0}-h_{1}\lambda \right)h_{1}\delta \lambda -\frac{h_{1}^{3}}{3}L_{1}L_{2}\rho \frac{d^{2}\lambda }{dt^{2}}\delta \lambda$$

where U is the applied voltage; *K* is the rigidity of the spring; *h*_0_ is the original length of the spring; *h*_1_ is the length of the compressed spring.

Inserting Equation (4) into Equations (3) and (5), we can obtain that

(6) $$\frac{K(h_{0}-h_{1}\lambda )}{L_{0}L_{2}}+\frac{U\varepsilon }{L_{0}^{2}}\lambda -\frac{L_{1}^{2}\rho }{3}\frac{d^{2}\lambda }{dt^{2}}=\sum_{i=1}^{3}\frac{\mu_{i}}{\alpha_{i}}\left(2\lambda_{}^{\alpha_{i}}-\lambda_{}^{-3\alpha_{i}}\right)$$

The relationship between the deformation rate and stress can be expressed as

(7) $$\frac{d\xi }{\xi dt}=\frac{\mu_{i}}{3\eta }(2\lambda^{2}\xi^{-2}-1-\lambda^{-2}\xi^{2})$$

where *η* is the viscosity of the dashpot; *ξ* is the stretch, due to the spring.

In order to achieve the bending deformation of the finger, half areas of the VHB are coated with electrodes. The relationship between $\lambda_{1}$, $\lambda_{1a}$, $\lambda_{1b}$ and *θ* can be expressed as

(8) $$\lambda_{1}=\frac{\lambda_{1a}+\lambda_{1b}}{2}$$

(9) $$\theta =\frac{\lambda_{1b}L_{1}-\lambda_{1a}L_{1}}{2r}$$

where $\lambda_{1a}$ is the pre-stretching ratio of the DE membrane in the concave, $\lambda_{1b}$ is the pre-stretching ratio in the convex, θ is the bending angle, and *r* is the curvature radius of the soft finger when the bending deformation is produced.

### 2.3. Finite Element Analysis of the Soft Finger

According to the above analysis of the soft finger, we build the finite element model. The model takes the DE membrane displacement and voltage in the soft finger as the independent inputs, and predicts the bending displacement as an output. For explicating analysis purposes, we focus our efforts on the static response of the soft finger and ignore the influence of its dynamic response (such as, the viscoelastic effects and snapping destabilization phenomena).

#### 2.3.1. Simulation of the DE Membrane

**Figure 3:**
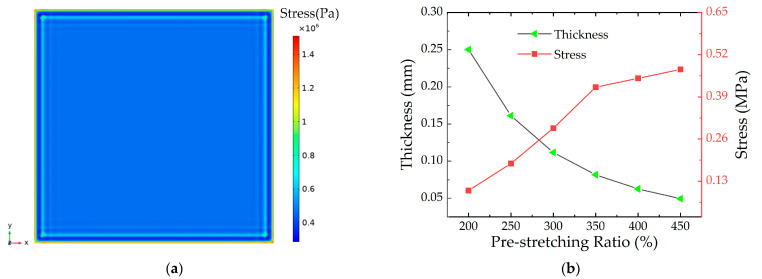
Stress cloud and data diagram. (**a**) Stress cloud of the DE membrane; (**b**) Thickness and center stress data of the DE membrane.

The specified displacement constraints are applied to the four boundaries of the model so as to simulate the equibiaxial pre-stretching effect. Figure 3 shows the stress distribution and the results of the pre-stretched DEs.

The stress distribution in the middle part is relatively uniform in Figure 3a. The stress concentration is on the boundary area of the DE membrane because of the drawing forces. Therefore, the middle part is taken as the experimental target and the stress is taken as the DE membrane stress. Because of the incompressibility of the DE, the thickness changes in the pre-stretching process. Figure 3b shows the simulations of the thickness and stress for the DE membranes at different pre-stretching ratios. It can be seen that the thickness of the DE membrane gradually becomes thinner with the increase of the pre-stretching ratio. At the same time, the stress of the DE membrane increases gradually with the increase of the pre-stretching ratio. When the pre-stretching ratio is λ = 400%, the thickness of the DE membrane is 0.0625 mm. Since its thickness is too thin, it is a significant influence on the electronic breakdown of the DE membrane. Therefore, the subsequent pre-stretching ratio of the DE membrane is between 300% and 350%, in this paper.

In order to simulate the pre-stretching effect of the DE membrane for a soft finger, a load is applied to one end of the model, and the load value is obtained through the tensile simulation analysis of the DE membrane. The initial model size of the DE membrane is 80 × 80 × 1 mm (consistent with the size of the VHB 4910), which is calculated by the Ogden model in Equation (4). The parameters are shown in Table 1.

#### 2.3.2. Boundary Conditions and the Simulation of the Soft Finger

The DE membrane is considered as a continuous hyperelastic body with electromechanical coupling and is subjected to a large deformation, so the simulation model is simplified, as follows: (1) The mechanical properties of the DE membranes are completely consistent, and there is no stress in the thickness direction. (2) The thickness and elastic modulus of the carbon paste, coated on the DE membranes, are ignored. (3) The carbon paste is regarded as an ideal dielectric material, which is subjected to an electric field, perpendicular to the surface of the DE membrane. (4) The corresponding boundary load is adopted at the end of the model to simulate the pre-stretching force applied by the DE membranes.

**Figure 4:**
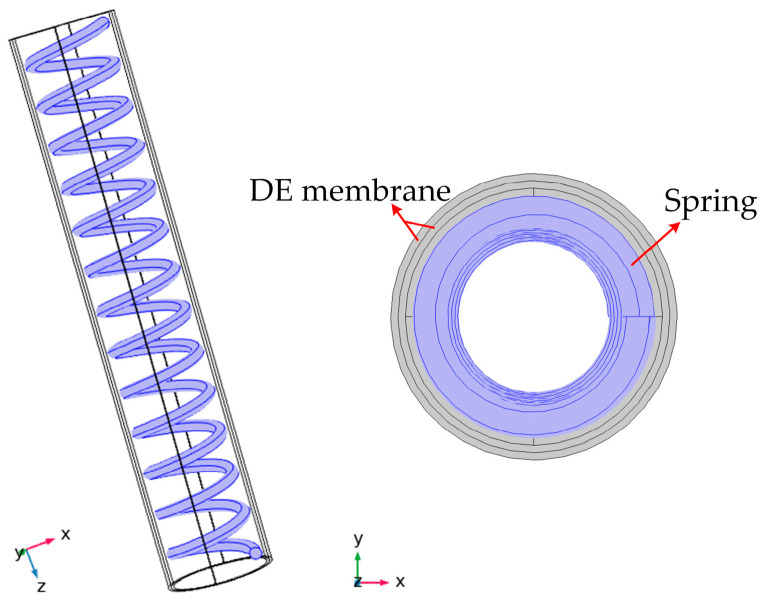
The model of a soft finger. The inner part is a rigid spring with a length of 80 mm and a wire diameter of 0.6 mm. The outer part consists of a two-layer DE membrane.

The models of the soft fingers are numerically processed in COMSOL Multiphysics. The built-in form of the Ogden hyperelastic free energy is modified by the parameters. In the paper, the rigid spring with an initial length of 80 mm and a wire diameter of 0.6 mm is used. The model of the soft finger is shown in Figure 4.

**Figure 5:**
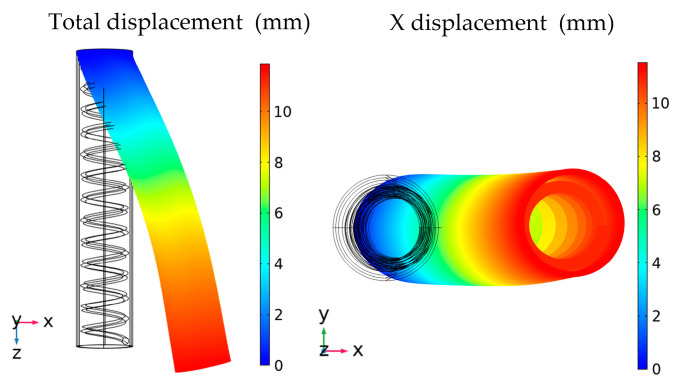
Simulation result of the soft finger. The displacement is 11.4 mm when the voltage is 4 kV.

The two-layer DE membranes are coupled with the spring by the connector command, and the electronic-mechanical module is used to calculate the deformation of the finger when the voltage is applied. The boundary condition is that, one side of the finger is fixed and the other is free. The calculated simulation deformation with the voltage of 4 kV is shown in Figure 5.

It is obvious that the bending deformation of the finger is obtained towards the direction without the electrode, and it is 11.4 mm in Figure 5. The result shows that there is an interaction between the DE and the spring. In addition, because of the hyperelasticity of the DE membrane, the finger also has a little elongation in the z-direction. According to the above simulation, there is an important impact of the bending deformation on the initial parameters, such as the compression of the spring, and the pre-stretching ratio of the DE membrane. In order to optimize the bending properties of the soft finger, the numerical analysis is carried out with these variable parameters.

#### 2.3.3. Numerical Analysis with the Different Compressions of the Spring

**Figure 6:**
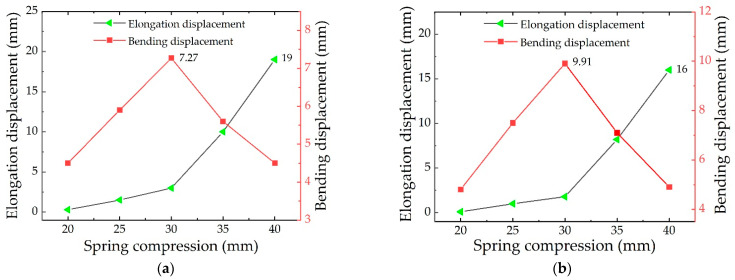
(**a**) the simulation results with the different compressions of the spring for a one-layer DE membrane. (**b**) the simulation results with the different compressions of the spring for a two-layer DE membrane.

In order to obtain the optimal compression of the spring, according to the simulation data of the DE membrane in Section 2.3.2, the pre-stretching ratio of the DE membrane is tentatively set to 350%. Considering that the spring is over-compressed (under-compressed), it leads to the elongation (shortening) phenomenon of the soft finger. Therefore, in order to reduce the secondary elongation (shortening), the range of the compression is 20–40 mm and five groups of data are analyzed by adding 5 mm in turn. Figure 6 shows the simulation results with different compressions of the spring for one-layer and two-layer DE membranes, respectively.

Figure 6 shows the change in the elongation displacement (the black curve) and bending displacement (the red curve) of the soft finger with the compression of the spring. From the black curves, it can be seen that when the compression of the spring is 20 mm, there is no elongation displacement. When the compression of the spring varies within 20–30 mm, the elongation of the soft finger gradually increases, but the change is small. When the compression of the spring exceeds 30 mm, the restoring force of the spring increases significantly, and the displacement significantly increases in the elongation direction. Because the number of layers is increased, the elongation displacement by the two-layer DE membrane is relatively smaller than that of the one-layer DE membrane.

It can be seen from the red curves that when the compression of the spring varies within 20–30 mm, the bending displacement also increases with the increase of the compression of the spring. When the compression of the spring is 30 mm, the bending displacement for the one-layer DE membrane and the two-layer DE membrane is 7.2 mm and 9.9 mm, respectively. However, when the compression of the spring exceeds 30 mm, the bending deformation suddenly decreases, due to the influence of the elongation deformation. Therefore, it is determined that the effective bending displacement of the soft fingers is the largest when the compression displacement of the spring is 30 mm, and the optimal compression displacement of the spring is 30 mm.

#### 2.3.4. Numerical Analysis with the Different Pre-stretching ratios of the DE Membrane

**Figure 7:**
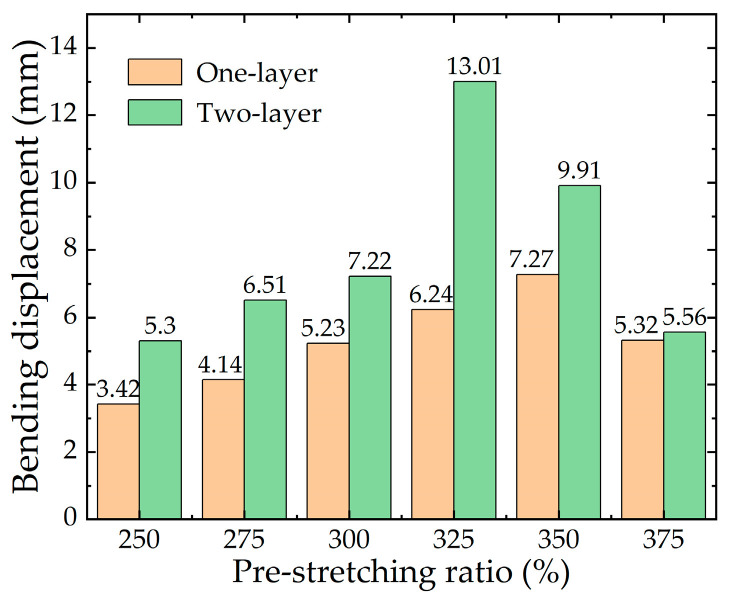
Simulation results with different layers and pre-stretching ratios of the DE membrane.

Figure 7 shows the simulation results of the soft fingers when the numbers of DE membrane layers are one and two, with different pre-stretching ratios of the DE membrane, respectively. It can be seen that, compared with the result for the one-layer DE membrane, the bending displacement for the two-layer DE membrane is significantly improved. When the pre-stretching ratio of the DE membrane is 325%, the bending displacement is increased by 6.77 mm. The data show that the bending displacement of soft fingers for the two-layer DE membranes is larger, which is more suitable for application in the soft gripper.

It can be seen from Figure 7, that when the pre-stretching ratio for the one-layer DE membrane is less than 350%, the bending displacement of the soft finger is positively correlated with the pre-stretching ratio of the DE membrane. When the pre-stretching ratio of the DE membrane is more than 350%, the bending properties of the soft fingers decrease. Combined with the data in Figure 3b, it is found that the thickness of the DE membrane becomes thinner when the pre-stretching ratio is more than 325%, which reduces the bending displacement of the soft finger. The maximum bending displacement of the soft finger with the one-layer DE membrane is 7.27 mm when the pre-stretching ratio is 350%. The maximum bending displacement of the soft finger with the two-layer DE membrane is 13.01 mm when the pre-stretching ratio is 325%.

Through the above simulation analysis of the soft fingers, it is found that the two variables, including the compression of the spring and the pre-stretching ratio of the DE membrane, have a significant influence on the bending performance of the soft finger on the same number of layers. The maximum bending displacement of a soft finger is 13.01 mm in the numerical range. In the following sections, the soft finger is prepared and experimentally tested to verify the rationality of the optimization design method.

## 3. Results and Discussion of the Soft Finger

### 3.1. Experimental Preparation of the Soft Finger

**Figure 8:**
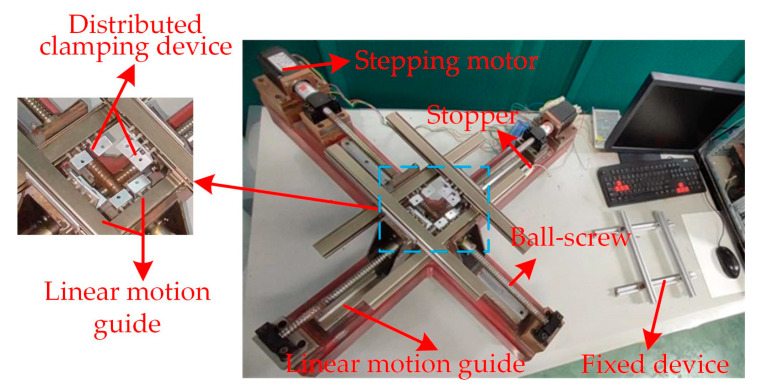
Experimental platform of the equibiaxial pre-stretching.

We research the experiments of soft fingers, and the experimental platform of the preparation for the fingers is built, as shown in Figure 8. The equibiaxial pre-stretching mechanism is provided.

**Figure 9:**
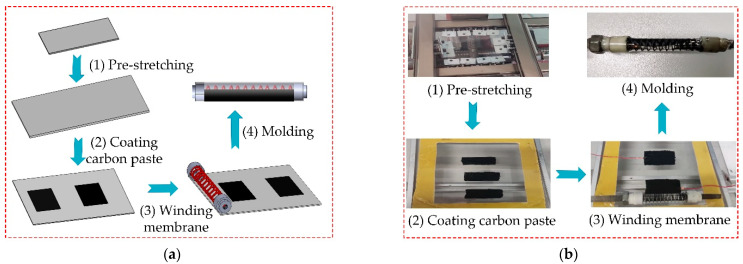
The production process of the soft finger. (**a**) The theoretical design process. (**b**) The experimental process.

The preparation process is designed in Figure 9a, and the experimental process is shown in Figure 9b. The production can be divided into four steps: (1) Pre-stretching the membrane; (2) Coating the electrode; (3) Winding the membrane; (4) Molding. Once the winding is completed and the limit nut is released, the compression force of the spring and the tensile force of the DE membrane reach a new balance position.

The detailed production steps are as follows:

(1) Pre-stretching the membrane: The initial DE membrane size is 7×7×1 cm, and the DE membrane is stretched to the corresponding size. The auxiliary frame is completely attached to the DE membrane.

(2) Coating the electrode: According to the maximum circumference and effective length of the soft finger, the carbon paste is internally coated, and the width and length of a single electrode coating (length 35 mm, width 20 mm) are calculated. In order to connect an external power supply, the copper foil and wires are put on the DE membrane.

(3) Winding the membrane: The compression of the spring is fixed by the cap and the nut, and the stress is maintained. The spring is put in the proper position of the auxiliary frame and the DE membrane is wound on the spring.

(4) Molding: The prepared unit is put out from the finger and the production of the finger is obtained.

### 3.2. Analysis of the Experimental Results

To verify the bending performance of the soft fingers, the experimental analysis is taken with the two variables, including the compression of the spring and the pre-stretching ratio of the DE membrane. The displacement of the soft fingers with different voltages is measured by adjusting the DC power supply.

#### 3.2.1. Experimental Analysis with the Different Compressions of the Spring

**Figure 10:**
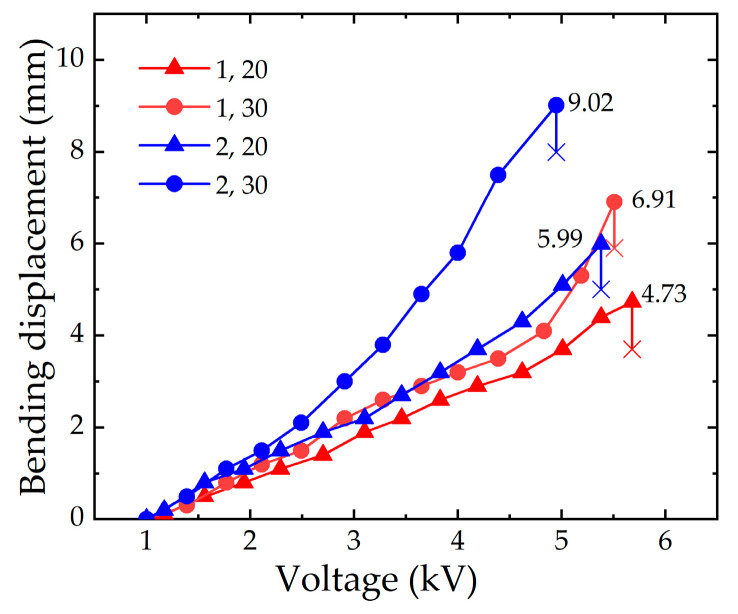
Experimental results of the different amounts of compression.

According to the simulation results of Section 2.3.3, the compression of the spring is 30 mm, and the pre-stretching ratio of the DE membrane is 350%. Experiments are carried out on the soft fingers when the numbers of layers are one and two, respectively. The experimental results are shown in Figure 10.

Figure 10 shows the variation trend of the bending displacement with the applied voltage for the different layers of the DE membrane. The red curves indicate that the maximum bending displacement of the soft finger with a one-layer DE membrane is 6.91 mm. The corresponding simulation result is 7.27 mm, and the error is 4.95%. The blue curves indicate that the maximum bending displacement of the soft finger with a two-layer DE membrane is 9.02 mm. The corresponding simulation result is 9.91 mm, and the error is 8.98%. The experiment results verify the effectiveness of the simulation.

#### 3.2.2. Experimental Analysis with Different Pre-stretching ratios of the DE Membrane

**Figure 11:**
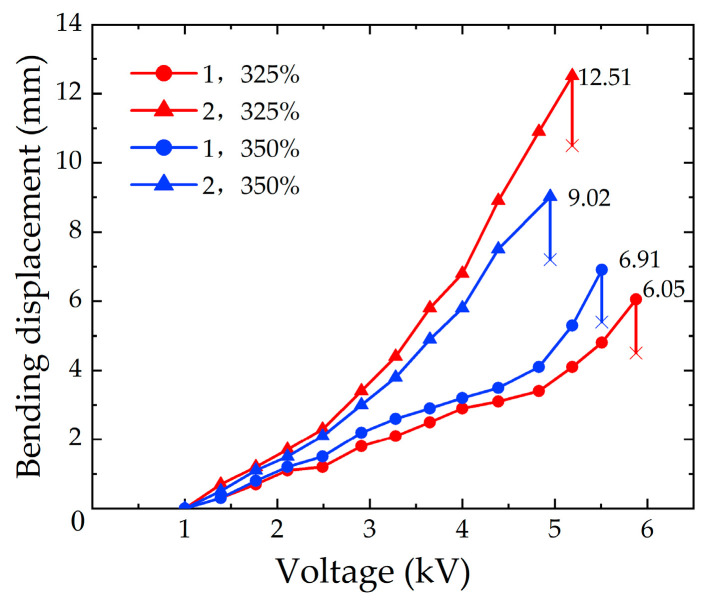
Experimental results of the different layers and the pre-stretching ratios of the DE membrane.

From the simulation results obtained in Section 2.3.4, the experiments are carried out when the pre-stretching ratios of the DE membrane are 325% and 350%, and the numbers of DE membrane layers are one and two, respectively. The results are as follows, as shown in Figure 11.

Figure 11 shows the variation trend of the bending displacement with the applied voltages. The two curves with the same color represent the same pre-stretching ratio of the DE membrane, and the two curves with the same graphic symbol represent the same number of layers of the DE membrane. It is obtained in the experiments that the maximum bending displacements of the soft fingers with a one-layer DE membrane are 6.05 mm and 6.91 mm when the pre-stretching ratios of the DE membrane are 325% and 350%, respectively. Compared with the simulation results of 6.24 mm and 7.27 mm, the errors are 3.04% and 4.95%, respectively. When the pre-stretching ratios of the soft fingers with the two-layer DE membrane are 325% and 350%, the maximum bending displacement are 12.51 mm and 9.02 mm, respectively. The errors with the simulation results of 13.01 mm and 9.91 mm are 3.84% and 8.98%, respectively.

The analysis of the experimental process shows that the breakdown voltage of the DE membrane has a significant influence on the bending displacement. When the pre-stretching ratio of the DE membrane is larger, the thickness of the DE membrane becomes thinner, and the breakdown voltage decreases. Therefore, the bending displacement becomes smaller. When the number of layers increases, the extrusion between the DE membranes affects the uniformity of the carbon paste. So it results in the decrease of the breakdown voltage, and the bending displacement is affected. In conclusion, within a certain range, the bending displacement of the soft finger is positively correlated with the two variables (the compression of the spring and the pre-stretching ratio of the DE membrane) on the same number of layers. With the continuous increase of the variable value, the bending performance of the soft finger is limited.

#### 3.2.3. Comparison of the Numerical Analysis and the Experimental Results

**Figure 12:**
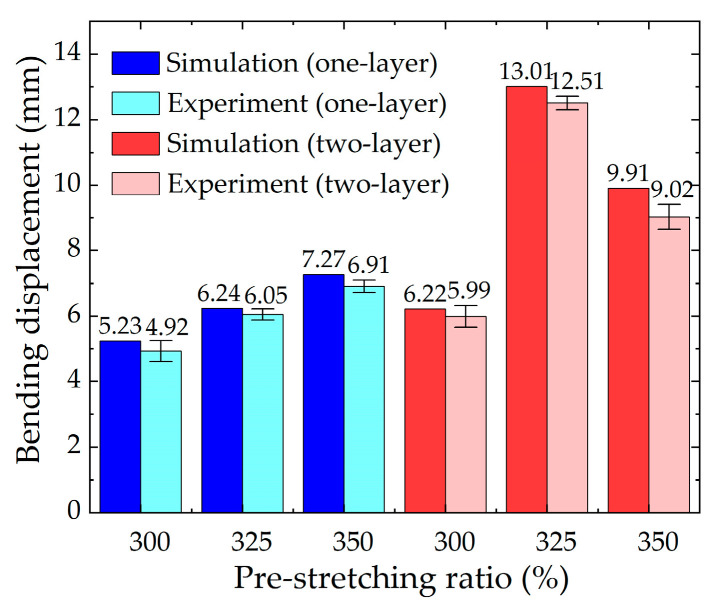
Numerical and experimental results of the soft finger with a one-layer DE membrane and a two-layer DE membrane, when the pre-stretching ratios are 300%, 325% and 350%, respectively.

Figure 12 shows the numerical and experimental results for the soft fingers. It can be seen from Figure 12 that the minimum relative error is 3.04% and the maximum is 8.98%. The maximum bending displacement of the soft finger with a two-layer DE membrane is 13.01 mm, in theory, and the bending angle is 20.39°, when the pre-stretching ratio is less than 325%. The relative error is 3.84%, compared with the experimental result of 12.51 mm and the bending angle is 19.66°.

Experiments verify that the data obtained by the rigid-flexible optimal design method proposed in the second section are basically consistent with the experimental results, and the bending deformation of the soft fingers is well predicted. The accuracy of the optimization design method proposed in this paper is verified.

**Figure 13:**
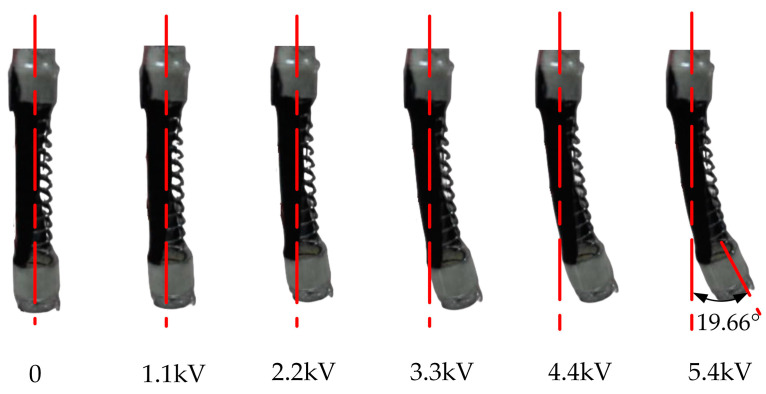
The deformations with the voltage from 0 to 5.4 kV (from left to right).

According to the above experiments, a set of optimal data is obtained: The output voltage can be adjusted between 0 and 5.4 kV by adjusting the voltage amplifier (EMCO Q101-5) and the DC power control valve. The maximum operating current is only 50μA after the amplification by the voltage amplifier, which will not cause a high risk. The bending deformation of the soft finger at different loading voltages is shown in Figure 13.

The soft finger obtained from the simulation and the experimental results can meet the requirements of the grasping objects. The soft gripper is prepared to achieve the target grasping with the above variable parameters.

### 3.3. Application of the Soft Finger

**Figure 14:**
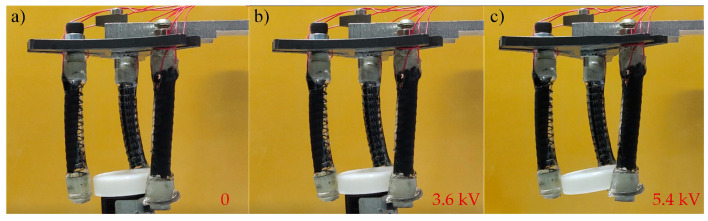
Grasping process of the soft gripper. (**a**) The condition when the voltage is 0; (**b**) The grasping condition when the voltage is 3.6 kV; (**c**) The final result of the grasping when the voltage is 5.4 kV.

According to the schematic diagram of the soft gripper structure in Section 2.1 (Figure 1), three identical soft fingers are produced and the gripper is manufactured with a weight of 13.04 g. The grasping experiments of the soft gripper are carried out on the experimental platform. The grasping process is shown in Figure 14a–c.

**Figure 15:**
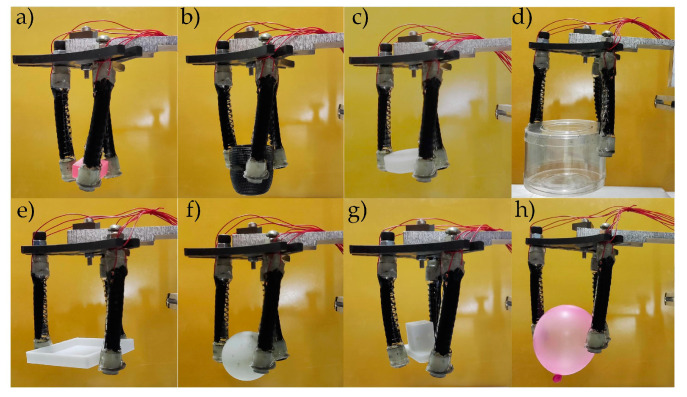
Grasping objects with different shapes: (**a**) cube (20 mm); (**b**) cylinder (24 mm); (**c**) cylinder (31 mm); (**d**) cylindrical box (60 mm); (**e**) cube (50 mm); (**f**) table tennis ball (30 mm); (**g**) irregular objects (20 mm); (**h**) balloon (55 mm).

In order to grasp objects of different sizes, the position of three soft fingers are flexibly adjusted. As illustrated in Figure 15a–d, the soft gripper could successfully grasp objects between 20 mm and 60 mm in size, including a 20 mm cube and a 31 mm cylinder with a voltage of 5.4 kV. With the power of voltage, the soft gripper could easily grasp cylinders, cubes, spheres and other shapes, respectively (Figure 15d–g). In addition, the soft gripper can not only grasp objects on hard surfaces, but also soft objects, such as balloons (Figure 15h), and the balloon is intact. According to the test, its maximum grasping quality is 11.21 g. Therefore, the soft gripper can grasp an extensive number of objects.

## 4. Conclusions

In this work, we explore the DE actuator as a flexible electroactive polymer into the soft finger and analyze the effect of the preparation parameters on the bending displacement of the soft finger. Therefore, we propose an optimization design method of the rigid-flexible soft fingers. The conclusions of this work are drawn as follows:

(1) Two parameters (compression of the spring and pre-stretching ratio of the DE) that have a significant influence on the bending displacement of the soft finger are obtained through a theoretical analysis. The rigid-flexible structure is analyzed through a numerical simulation and the optimal values of the two parameters are obtained with the one-layer and two-layer DE membranes, respectively. The bending displacement of the soft finger has been a significant improvement.

(2) The effectiveness of the proposed method is proved by the experiments, and the minimum relative error is 3.04% and the maximum is 8.98%. It could also provide a reference for the optimization design of soft grippers and soft robots.

(3) We assembled three soft fingers into a soft gripper with which various objects were grasped, and the weight of the grasped object reached 86% of its body weight, and we demonstrated the soft gripper’s capability to grab objects with its fingertip. The ratio between the power input and output was 70%.

The proposed method is suitable for rigid-flexible structures and can significantly improve the bending performance of the soft finger, and could be applied to optimize the mechanisms and soft robots. Based on this research, future work will focus on analyzing the grasping force of the fingertip and improving its grasping capability with a better rigidity.

## Figures and Tables

**Table 1:** Ogden third order hyperelastic material model parameter table.

Property	Value	Unit
Shear Elasticity μ_1_	24,940	Pa
Shear Elasticity μ_2_	57,340	Pa
Shear Elasticity μ_3_	−57,400	Pa
Parameter α_1_	1.2814	-
Parameter α_2_	3.0164	-
Parameter α_3_	3.0158	-

## Data Availability

The data presented in this study are available upon request from the corresponding author.
